# Nervous system modulation through electrical stimulation in companion animals

**DOI:** 10.1186/s13028-021-00585-z

**Published:** 2021-05-30

**Authors:** Ângela Martins, Débora Gouveia, Ana Cardoso, Óscar Gamboa, Darryl Millis, António Ferreira

**Affiliations:** 1 Animal Rehabilitation Center, Arrábida Veterinary Hospital, Azeitão, Setúbal Portugal; 2 Faculty of Veterinary Medicine, Lusófona University, Campo Grande, Lisboa, Portugal; 3 Faculty of Veterinary Medicine, University of Lisbon, Lisboa, Portugal; 4 Department of Small Animal Clinical Sciences, University of Tennessee College of Veterinary Medicine, Knoxville, TN USA

**Keywords:** Cat, Dog, Electrostimulation, Functionality, Neurorehabilitation modalities, Spinal cord

## Abstract

Domestic animals with severe spontaneous spinal cord injury (SCI), including dogs and cats that are deep pain perception negative (DPP−), can benefit from specific evaluations involving neurorehabilitation integrative protocols. In human medicine, patients without deep pain sensation, classified as grade A on the American Spinal Injury Association (ASIA) impairment scale, can recover after multidisciplinary approaches that include rehabilitation modalities, such as functional electrical stimulation (FES), transcutaneous electrical spinal cord stimulation (TESCS) and transcranial direct current stimulation (TDCS). This review intends to explore the history, biophysics, neurophysiology, neuroanatomy and the parameters of FES, TESCS, and TDCS, as safe and noninvasive rehabilitation modalities applied in the veterinary field. Additional studies need to be conducted in clinical settings to successfully implement these guidelines in dogs and cats.

## Background

Locomotion requires the coordination of movement and extensor/flexor muscles in all limbs. This coordination is due to the partial influence of all descending motor tracts in humans as well as dogs and cats [1].

In humans, the influence of the corticospinal tract is greater than in dogs and cats. The corticospinal tract is less developed in domestic animals, especially dogs, resulting in difficulties to perform complex and precise movements [2, 3].

Bipeds, including humans, and quadrupeds share many similarities. They have the same major descending motor tracts, however with the pyramidal system predominating in humans as opposed to dogs and cats, where the extrapyramidal system predominates [4–6]. In humans, the rubrospinal descending motor tract is considered to be vestigial in humans [7, 8], but this is of major importance in domestic animals. In dogs, this tract is considered the main tract that controls voluntary movement, as it can facilitate action of the lower motor neurons (LMNs) in flexor muscles [4]. Moreover, in cats, apart from rubrospinal tract influence, the corticospinal tract has a participative role in precise and complex movements [9].

The reticulospinal tract (RST) plays a prominent role in the motor and postural control, which has been well demonstrated in cats. Additionally, a balance between pontine and medullary RST is essential for locomotion, posture and muscle tone [9, 10]. The pontine RST facilitates spinal motoneurons (α and γ) for the extensor muscles, and the medullar RST inhibits spinal motoneurons (α and γ) for the extensor muscles and simultaneously facilitates motoneurons for the flexor muscles [3, 4, 9] in both species.

For neurorehabilitation, the reorganization of these tracts derived from reticular formation is essential to promote spinal reflexes, motor activity and postural standing, as these tracts play an important role in muscle tone [9].

There are some neurophysiological differences between dogs and humans; for example, in humans, the RST is composed of the dorsal and medial RSTs [11]. However, in the two species, the propriospinal tracts share similar spinal neural control mechanisms [12] and exhibit similar coordination patterns between limbs during locomotion [13].

Neurorehabilitation training for dogs and cats that are deep pain perception positive (DPP+), as well as for human patients who have American Spinal Injury Association (ASIA) impairment grades of B, C, or D [14], can improve functional gait after injury by improving coordination, balance, and muscle strength and shortening the recovery time [15, 16].

Thus, functionality can be defined in a similar way as ‘ambulatory’, describing the ability to stand up, maintain active postural balance during standing and take at least ten consecutive active, weight-bearing steps [3] without falling. Impairments in functionality may be related to proprioceptive deficits.

Dogs and cats classified as DPP+, as well as human patients with ASIA B/C/D spinal cord injuries (SCIs), are considered to have an incomplete SCI. Therefore, it can be beneficial to stimulate descending motor spinal cord pathways and afferent inputs that have branches connecting to central pattern generators (CPGs). CPGs are connected to propriospinal neurons, the majority of which are interneurons connecting multiple segments that can control LMNs, and re-establish basic gait motor rhythmicity [17, 18].

In humans with subacute and chronic motor incomplete SCI, different approaches can be considered to provide assistance during stepping [19], such as manual locomotor training (body weight-supported treadmill training or overground training) and robotic devices [20]. Additionally, in different studies, locomotor training has been performed with functional electrical stimulation (FES) [21–24].

In both human and veterinary medicine, permanent deep pain perception negative (DPP−) can be an indication of a complete spinal cord injury [25, 26].

In cats classified as DPP−, repetitions of the same movement and the associated sensory feedback that occurs from that movement may induce the essential plastic adaptations of network neurons. Thus, rehabilitation modalities can be useful tools and require the involvement of not only network neurons but also some residual supraspinal pathways [27].

In dogs with severe SCI, DPP should be evaluated for 24 h in the event that the clinical presentation changes [28–30]. The most severe injuries are related to paraplegic DPP−. A previous study, in DPP− dogs, showed a recovery rate of 61% after surgical decompression and 10% after conservative treatment [31]. Thus, DPP assessments are performed to determine whether there is a conscious response to a mechanical stimulus in the medial and lateral digits of both forelimbs and hindlimbs, the tip/base of the tail and the perineal region (scrotum/vulva and anus). This assessment is essential as a prognostic indicator.

Incomplete to complete SCIs involve primary and secondary injuries. The primary injury is usually caused by a relative contribution of both compressive and contusive forces caused by structures anatomically located ventral to the spinal cord [32]. Concussion manifests in a more severe form, and dogs are usually paralyzed with absent DPP [13, 33]*,* which indicates an extremely poor prognosis for functional recovery [34].

The secondary SCI are caused by biochemical and metabolic damage 2 to 48 h postinjury. At this time, there is a massive release of glutamate and other central excitatory neurotransmitters, which promote excitotoxicity [35], as well as oxidative damage and inflammation [36].

If DPP is lost, the spinoreticular tracts, propriospinal tracts, and possibly even the spinothalamic tracts near the spinal cord gray matter can be affected [37, 38].

In clinical settings, a relation between the presence of DPP and ambulation has been examined. Thus, paraplegic dogs with intact DPP are more likely to regain the ability to ambulate when compared to DPP− dogs [39]. In these cases, the ability to locomote is dependent on the neural reorganization of motor descending tracts and sensory inputs [3].

Deep pain perception negative dogs and cats, as well as human patients, can exhibit functional recovery through neurorehabilitation, given the similarities between the three species [40–42]. These multidisciplinary treatment protocols are based on a group of neurorehabilitation modalities, such as FES, transcutaneous electrical spinal cord stimulation (TESCS) and transcranial direct current stimulation (TDCS).

Neurorehabilitation modalities can be useful for stimulating neurogenesis and strengthening the pre-existing neural tracts [43] that promote anatomic and synaptic neuroplasticity [5], both cranial and caudal to the injury site and possibly through it. Spontaneous reorganization of the motor system leads to the sprouting of spared fibers of the RST [44, 45].

Previous researchers have focused on neural modulation and have studied reflexes and their roles in improving functionality and generating flexion–extension locomotion patterns [46]. The therapeutic potential of neural modulation can be illustrated by TESCS, which promotes the maintenance of axonal connections in severe SCI when massive destruction of the spinal parenchyma occurs, especially in trauma patients [47].

This review article intends to explore the history, biophysics, neurophysiology and parameters of the following neuromodulation modalities: FES, TESCS, and TDCS.

## Review

### Functional electrical stimulation as a neurorehabilitation therapeutic modality

Functional electrical stimulation is a general tool used for all applications targeting the activation or restoration of function by electrical stimulation, including efferent and afferent nerve stimulation and neuromuscular and muscle stimulation [48].

This neuromodality uses sequences of short bursts of electrical pulses to stimulate the LMNs near the motor endplate region or through peripheral afferent nerves, resulting in the activation of a peripheral spinal reflex [49, 50].

FES was applied for the first time by Liberson et al. [51], who used two superficial electrodes to stimulate the peroneal branch of the sciatic nerve of human patients who exhibited unilateral weakness following a stroke.

Furthermore, FES was used in human patients with SCI to initiate a motor response and restore lost function by the stimulation of the quadriceps group during the stance phase and the end of the swing phase. Additionally, with the stimulation of the peroneal nerve in the popliteal fossa region and the stimulation of the saphenous nerve, the results showed a flexion withdrawal response producing movement in the swing phase [52].

To obtain that response, the current administered by FES is a low intensity current but sufficient to trigger an action potential to induce a visible muscle contraction [53]. In some cases, this stimulation can be performed by placing the electrodes near the muscle mechanoreceptors [34, 54].

To prevent excessive neuromuscular fatigue, it is important to select the correct electrical parameters, including the current magnitude, pulse amplitude, total treatment time, current frequency, current waveform and duty cycle [55].

FES induces the unnatural recruitment of muscle fibers, mostly by recruiting large-diameter motor neurons, which are considered to have fast conduction velocity fibers, instead of recruiting small-diameter motor neurons, which are slower and fatigue resistant [56].

**Fig. 1:**
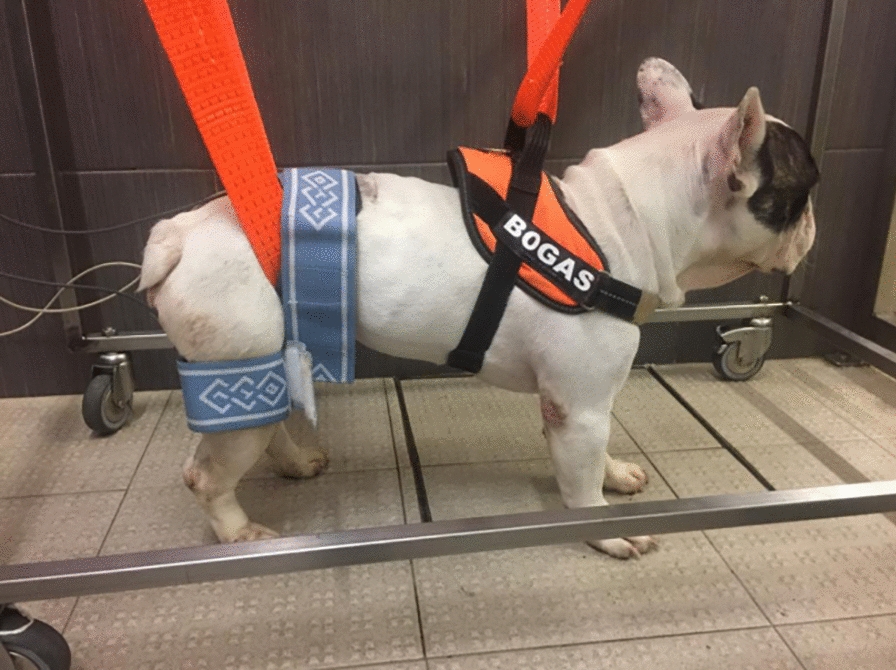
Functional electrical study—Application of segmental FES modality on a dog

**Fig. 2:**
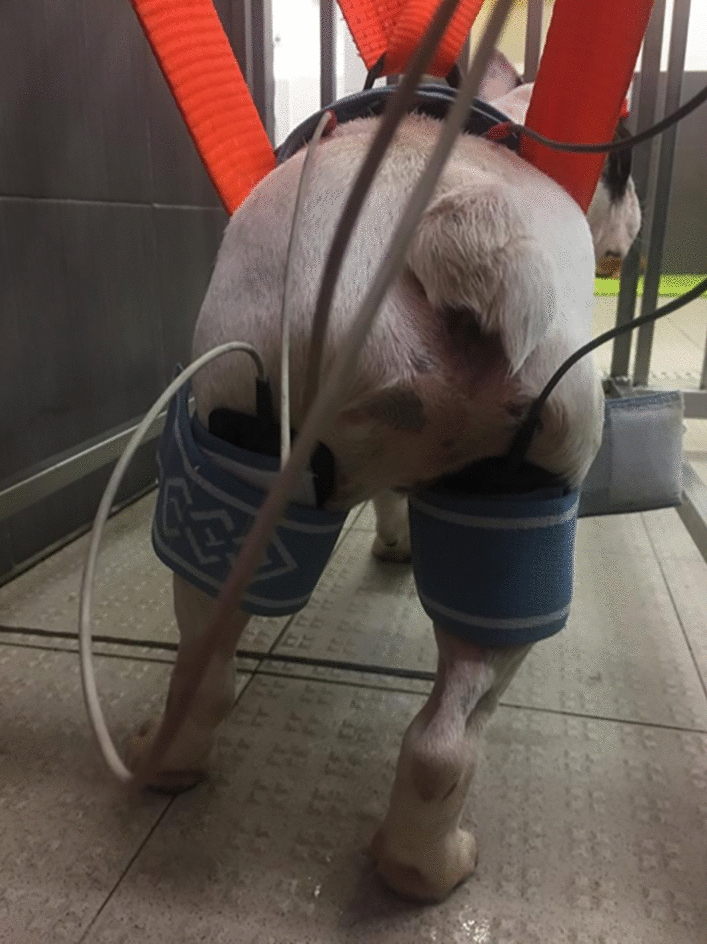
FES protocol to promote new connections—Application of the anode near the motor point region

On this basis, using superficial electrodes, FES is used to strengthen muscles, improve blood flow and avoid progression of muscle atrophy (Figs. 1, 2). Additionally, FES can electrically stimulate noninnervated tissue and paralyzed and spastic muscles [57, 58]. To decrease discomfort, in dogs, a short pulse duration as low as 20 Hz is usually recommended for a tetanic muscle contraction. However, the maximal force of contraction generally occurs between 60 and 100 Hz (more commonly in noninnervated tissue). Thus, a lower tetanic frequency within the range of 25–50 Hz and a lower intensity, but over 2.5 mA, will minimize fatigue (more commonly in innervated tissue) [59].

Patients with severe atrophy (neurogenic atrophy) may need longer off-time to recover, starting with duty cycle ratios of 1:5, and these patients should be monitored for signs of fatigue. Additionally, a ramp-up time of 2–4 s and a ramp-down time of 1–2 s should be considered [59].

Since 2012, in the field of restorative neurology, FES has been considered a possible tool for achieving a functional ambulatory state by activating spinal interneuron intrinsic circuits and CPGs that are involved in both voluntary and spinal reflex locomotion [60, 61].

FES allows long-latency flexor reflex development in humans with complete SCI, which has been compared to the late flexor reflex observed in animals with the same injury. This reflex in cats is considered to utilize interneurons of spinal locomotor centers that are associated with afferent stimulation [62], generating the flexor reflex and the swing phase of stepping [63].

Thus, FES protocols can promote the excitability of new connections, which can be created during the process of neural reorganization as a result of rehabilitative programs. Moreover, it is known that these programs are more effective when they are implemented only a few days after spinal injury [20].

FES is considered a noninvasive modality that is easy to perform in a clinical setting and can be used for neuromodulation.

The combination of FES and locomotor training protocols has been shown to have therapeutic benefits among both animals and human patients [64, 65], which indicates that these protocols affect neuroplasticity and functional locomotion, particularly in regard to speed, step length and cadence [64]. It has also been proposed that FES can facilitate or enhance reciprocal inhibition, which is mainly mediated by interneuron activation through Ia fibers on the contralateral side [66].

### Transcutaneous electrical spinal cord stimulation as a neurorehabilitation therapeutic modality

Transcutaneous electrical spinal cord stimulation is a therapeutic method of electrically activating spinal cord circuits by means of surface electrodes placed on the skin in the cervical, thoracic and lumbosacral regions [67].

TESCS is a noninvasive and nonpainful neuromodulation modality that induces stimulation throughout synergistic and interactive multisegmental paths, combining the central components of motor descending tracts and ascending sensorial tracts. Thus, it may recruit a different population of motor neurons by projecting sensory and intraspinal connections, directly resulting in the amplification of spinal evoked motor potentials [68]. Multifocal TESCS may have a role in human locomotor activities that involve the monosynaptic neural circuitry of the extensors and flexors of the knee and ankle and in achieving unassisted standing with correct weight distributions [69].

Lloyd [70] was the first to investigate electrical stimulation through the dorsal and ventral horn of the same spinal segment, maximally inducing the spinal peripheral reflex in the transmission between the two neurons, which was named the ventral horn/muscle reflex (RCV/M).

Minassian et al. [71] performed a study to demonstrate the action of TESCS with electrodes (cathodes) placed over the T11-T12 vertebral region and the anode on the umbilical region to create two channels. This stimulation through surface electrodes allowed perpendicular current penetration, migrating the ligaments between the spinous processes in the vertebral lamina, penetrating the vertebral canal and promoting a current in the cerebrospinal fluid, with the purpose of stimulating the RCV/M from the quadriceps femoris, hamstrings, tibial anterior and triceps surae. The results provided evidence that TESCS can depolarize the lumbosacral posterior roots.

Kitano and Koceja [72] showed that the monosynaptic multisegmental response of TESCS is superior to that of traditional peripheral electrical stimulation, with a maximum RCV/M or H-reflex, because TESCS allows selective excitation of the sensorial fibers, thereby producing a direct motor response and an antidromic current on the motor fibers.

Many authors studied the T11-T12 anatomical region, as previously described. However, for other authors, maximal stimulation with TESCS was best performed with the electrodes placed in the T12-L1 intervertebral region [71–76]. However, Akaza et al. concluded that the placement of the electrodes should be determined on an individual basis [76].

According to Roy et al. [77] the best location to stimulate a large number of connections in the lumbosacral intumescence with biphasic current was considered to be T11-T12 or T12-L1. However, for the same authors, the position of the cathode along the vertebral column was of greater importance, and the authors noted that sensory neural fiber stimulation is supported by the L1-L3 region; however, for the recruitment of motor neuron fibers, the best neuroanatomical location was caudal to the L5-S1 anatomical region.

Regarding the anatomical region of electrode placement in humans, geometric changes in the thoracolumbar vertebral column, as well as the position of adjacent vertebra, can influence the progression of current depolarization. Therefore, the stimulation of interconnections at the level of the lumbosacral intumescence may be affected [77].

Hofstoetter et al. [78] placed electrodes at the standard location of T11/T12 and applied a symmetric biphasic continuous current of 50 Hz, intending to depolarize the large-diameter afferent fibers of the L2-S2 neuroanatomical region and its posterior roots in humans, and the authors concluded that the activated connections were superior to those of the epidural technique [79]. In 2012 the same authors used a biphasic continuous current with a range ≤ 100 and observed contractions of the paraspinal and abdominal muscles, in addition to paraesthesia in the lower limbs in bipedal patients, which was expected [61].

Currently, in human medicine, the application of this modality leads to a decrease in muscle contraction coactivation, leading to a faster gait in bipedal patients. This outcome was observed in three patients; decreases in coactivation during the tonic and clonic phases in peripheral flexor reflex examinations and thus a decrease in muscle spasms were observed [61, 78, 79].

As mentioned before and confirmed by different authors, the current enters the spinal canal, but only a small portion, approximately 8%, of the current enters the canal [80]. The current depolarizes the nerve root because it progresses through the motor descending spinal cord tracts until it reaches the neural intrinsic circuitry of the lumbosacral region, normally preserved caudal to the injury [79]. This event can be assessed by visual vibrations on the Achilles tendon and the presence of passive or active contractions at the level of the lower limbs in humans [78]. By our own experience, this event also occurs in dogs and cats.

Hofstoetter et al. [81] showed that motor descending tracts can be depolarized by the spinal cord-brainstem-brain-spinal cord loop (SBBSL) phenomenon [82]. The stimulation of afferent nerves of the forelimbs in quadrupedal animals directly influences pelvic limb activity by activating proprioceptive descending tracts. Therefore, afferent stimuli cross the spinothalamic and spinocerebellar tracts to the locomotor region of the brainstem or the motor cortex in humans. Moreover, through descending bulbospinal tracts, it is possible to activate the spinal network of the lumbosacral intumescence.

In human patients with spasticity, when electrodes are placed below the level of the lesion, TESCS can promote temporary anti-spastic effects through nonspecific inhibitory mechanisms [83]. The most commonly used frequencies for neural stimulation are approximately 100 Hz and lower [84–86]. However, to control spasticity, the frequency should range between 50 and 100 Hz [87] because stimulation at higher frequencies can not only electrically activate the same neural input structures but also lead to maximum action potentials.

For locomotor function, approximately stimulation at frequencies of 20 to 50 Hz should be used in patients with SCI [88, 89].

Simulation at frequencies within this range can evoke action potentials of the descending motor tracts and stimulate Ia afferent fibers that have a strong synaptic connection to Ia inhibitory interneurons (reciprocal inhibition mechanism) while avoiding paresthesia, allowing muscle contractions, and reducing the risk of spasticity [82].

According to different authors, long propriospinal neurons play a key role in the functional connection between the cervical and lumbosacral regions and allow coordination between limbs. Locomotor coordination depends on the propriospinal system, which can be activated by the stimulation of limb peripheral nerves in the thoracic and cervical regions [90, 91].

Therefore, multisegmental TESCS (MS-TESCS) with electrodes placed at C5-C6, T11-T12, and L1-L2 promotes propriospinal system neuromodulation and thus gait cycle coordination [92, 93]. Some studies used the same modality with a 5–40 Hz biphasic current that could reach 30–200 mA while maintaining the severity of pain below a predetermined threshold [94, 95].

MS-TESCS enables the convergent multisegmental stimulation of ascending and descending spinal cord tracts, which is associated with propriospinal system neuromodulation and positively influences spinal intrinsic circuits and triggers locomotion. MS-TESCS is a noninvasive neuromodulation modality that is used in human patients, and it can be considered a potential treatment option for DPP+ or DPP− paraplegic dogs [94].

TESCS, with a 50 Hz biphasic current in a 30-min session, has been proposed to be a viable nonpharmacological treatment [96]. This type of current has a rectangular ramp that helps control and improve patient comfort, with a ramp-up time of 4 s, ramp-down of 2 s and plateau close to 10 s [59]. In the study, the amplitude was increased until lower limb paresthesia was present, ranging from 15 to 90 mA. These stimulations can induce spinal reflexes in the lower limbs. Within 2 days, consecutive TESCS demonstrated moderate-to-high reproducibility of the spinal reflex recruitment properties [97].

It is important to mention the human randomized, sham-controlled, double-blinded, parallel design study published by Awosika et al*.* [98] as the results indicated that anodal TESCS may be related to the neurorehabilitation of locomotor function after neurological injury. Anodal TESCS affected speed and symmetry, as well as flexion/extension locomotor pattern modulation, and increased blood flow to the spinal cord, especially when TESCS was repeated daily.

Ammendolia et al*.* [99, 100] showed an increase in blood flow to the spinal cord and cauda equina with TESCS, and the magnitude of effect was dependent on the intensity of the electrical stimulus.

Cervical TESCS has also been shown to improve the voluntary control of hand function in tetraplegic patients [101], although the neurophysiological mechanisms are still poorly understood. The amplitude of the current used ranged from 50 to 90 mA, and there were evident similarities with lumbosacral TESCS [102].

The spinal neural mechanism is mostly based on both afferent sensory stimuli and the neural intrinsic circuitry pattern, which are present in both humans and dogs [103]. Although there is no literature regarding TESCS applied in small animals, it is possible to translate the therapeutic potential of this modality to veterinary patients, considering all neuroanatomic differences.

The intrinsic circuitry pattern and CPGs constitute the neural network located in the intumescence in both the brachial and lumbosacral plexuses. However, in dogs, the lumbrosacral plexus is located in L4-S3 (spinal cord segments corresponding to L3-L7, S1-S3, Co1–Co 3 or more) [4, 104], and in humans, it is located in L2-L3, with the spinal cord ending at the inferior side of the L1 vertebrae [105].

**Fig. 3:**
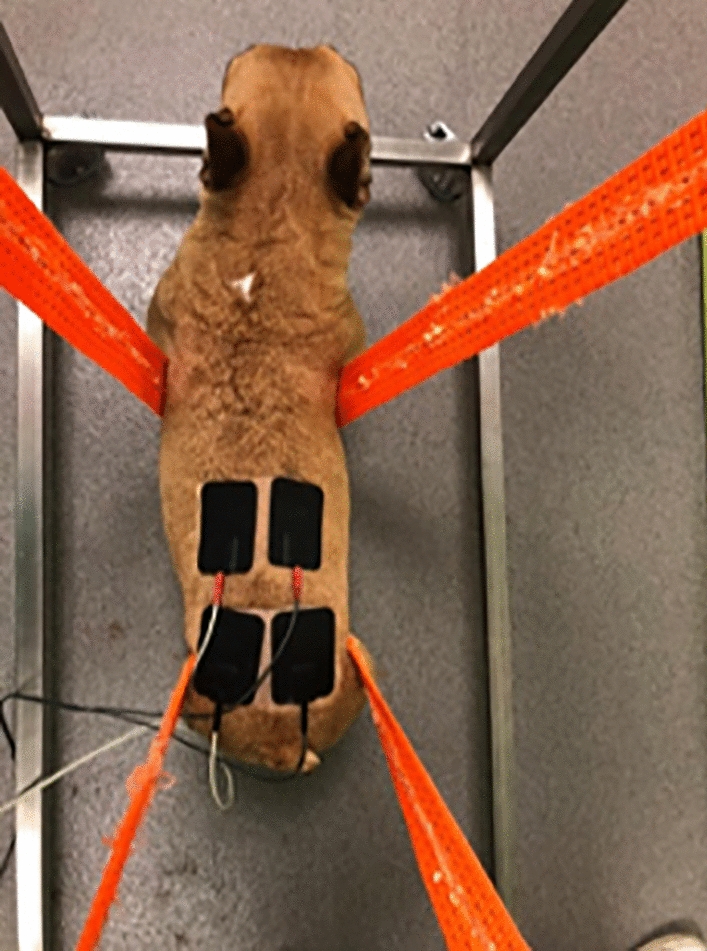
Transcutaneous electrical spinal cord stimulation—Application of L2-L3 TESCS modality on a dog

**Fig. 4:**
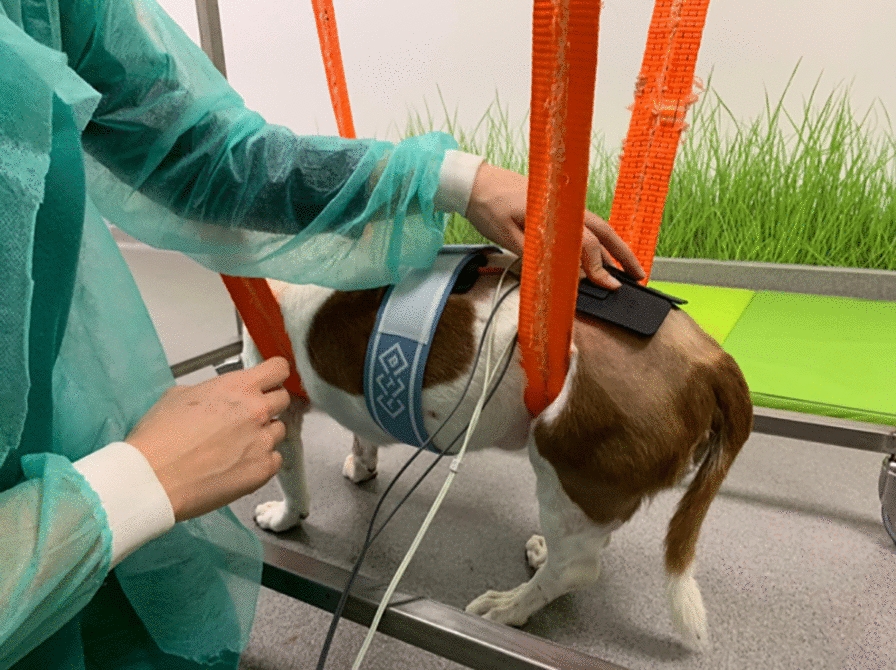
Transcutaneous electrical spinal cord stimulation—Application of L2-L3 TESCS, according to the vertebral column geometric anatomy

Therefore, for translational medicine, in dogs, the cathode should be placed in the L2-L3 region, that is, at the beginning of the pelvic intumescence. The anode should be placed on the dorsal edge of the iliac crest, corresponding to the spinous process of L7 (Figs. 3, 4).

### Transcranial direct current stimulation as a neurorehabilitation therapeutic modality

Transcranial direct current stimulation is a method of noninvasive transcranial stimulation that uses a weak direct current [< 1.5 milliamperes (mA)], which is not painful and easy to apply [95, 106].

Studies have demonstrated that TDCS is responsible for motor cortex-modulated excitability in humans [107–109]. This excitability is maintained for a few minutes after TDCS application, which allows interconnections to be created not only at anatomical region at which the electrodes are placed but also in other regions [110–112].

This neuromodality has been used in stroke patients since 2005 to promote motor deficit recovery [113] and in patients with neuropsychiatric diseases and chronic neuropathic pain [114–116].

Previous studies have demonstrated the use of TDCS in neuropathic pain management through A delta fiber activation (as well as A beta fibers and sometimes C fibers) and through the spinothalamic pathway [117] with the use of 2 and 2.5 mA currents for 15–20 min, respectively [118].

The number of stimulation sessions, frequency, intensity and stimulation region are important factors of treatment to consider [119]. Sessions repeated every 24 h have higher efficacy than do single sessions due to the cumulative effect, and the effects are expected to last longer when TDCS is applied over longer periods of time, including a period of 2 weeks [120].

**Fig. 5:**
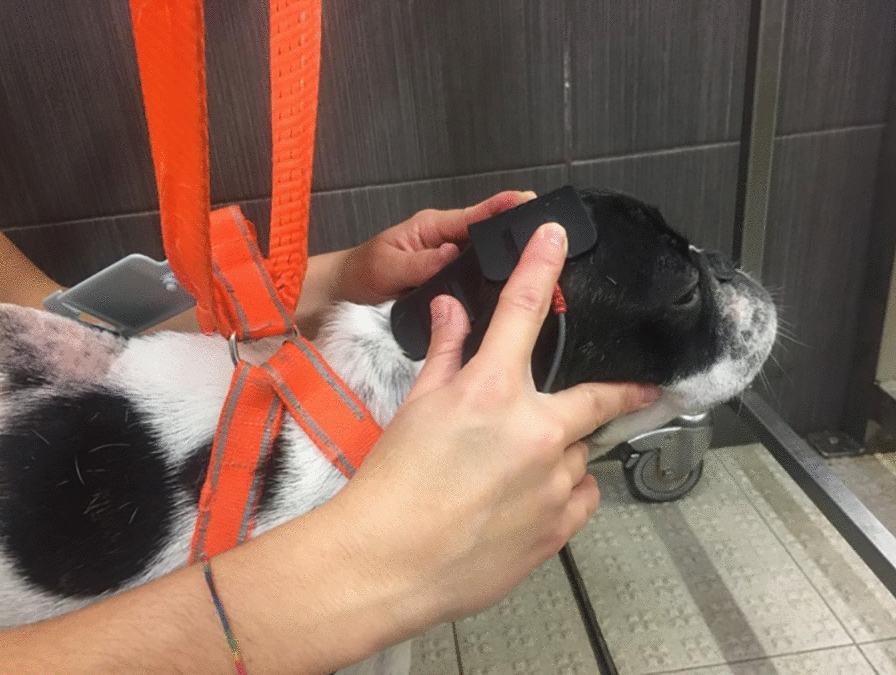
Application of TDCS modality on a dog—Anode localization near the brainstem region

The characteristics of a neural action potential depend on the cathode and anode placement and the current of the electrodes. The cathode contributes to neuronal hyperpolarization and consequently to a reduction in neuronal excitability (Cathode TDCS), and the anode is responsible for the desired neuronal depolarization, allowing neuronal excitability to take place (Anode TDCS) [115, 116] (Fig. 5).

Thus, TDCS success depends on the interaction between the electrical current and geometrical parameters [121]. Stecker [8] found that cranial stimulation is a complex phenomenon that depends not only on cerebral geometry and the conductivity of intracranial membranes but also on the complex skin and skull geometry, the cerebrospinal fluid and the brain, which are associated with the anisotropic conductivity of the different structures and their variability among patients [111].

TDCS effects can persist for five hours or sometimes more when the stimulation is applied for 10 to 30 min [7]. According to different authors, whenever TDCS is applied for longer than 10 min, with a current close to 1 mA, the effects persist for at least one hour [113, 115].

**Fig. 6:**
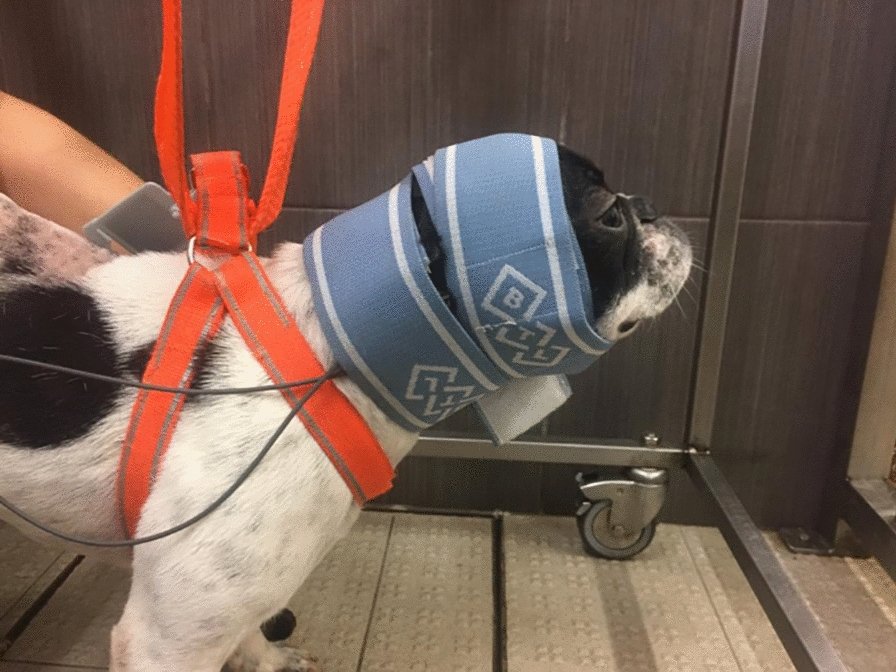
Transcranial direct current stimulation—TDCS protocol based on a 1 mA current for 20 min on a dog

The standard range of the current is usually between 1 and 2 mA, and currents within this range lead to smaller peripheral side effects than do larger currents [122, 123] due to the electrochemical production of toxic substances at the electrode interface, which has been observed both in rats and in humans [115]. Additionally, this type of electrical current does not intend to achieve muscle contraction however can have some cumulative effects. However, the existing information on TDCS current intensification is limited [115, 122]. Thus, standard TDCS protocols performed in humans usually use 1 mA pulsed currents ramped up and then down over 30 s windows and 35 cm^2^ and 25 cm^2^ electrodes [106, 113, 124], which are chosen to minimize the risk of unintended effects [125]. This stimulation can be applied for a duration of 20 min for 5 consecutive days [125] (Fig. 6).

A study in rats used currents of up to 10 mA to increase cerebral cortex-modulated excitability, and there was no evidence of thermal secondary effects or neurotoxicity. However, additional studies need to be conducted to determine the morphological long-term changes and the therapeutic effects of intensifying protocols without causing harmful effects [115].

In 2000, the neurophysiological mechanism of TDCS was presumed to be associated with motor cortex excitability, which can induce changes in spinal cord excitability [110]. TDCS induces changes in cerebral excitability by up to 40%, likely due to functional synaptic mechanisms that are dependent on current intensity and stimulation duration [126].

In 2003, research suggested that the neurophysiological effects of TDCS are caused by ionic alterations at the cellular level, which lead to increased intracellular Ca^2+^ concentrations. Additionally, in 2005, the same author suggested that TDCS can improve excitability-related changes in plasticity [95, 109, 127]. Thus, recent results from animal studies show that intracellular calcium Ca^2+^ concentrations determine the magnitude of increase/decrease in synaptic connections and that the strength of plasticity depends on the amount of calcium influx. Long and periods of Ca^2+^ influx cause the depression of axonal synapses, and moderate periods of calcium influx do not induce synaptic modulation, whereas a large Ca^2+^ influx increases the potentiation of synaptic modulation [128]. Additionally, glutamatergic activity with N-methyl-d-aspartate (NMDA) receptors, which have Ca^2+^ channel properties, has been proposed to be a possible mechanism of induced plasticity [129]. In vivo animal studies have revealed that the TDCS current impacts the neuronal firing rate, modifying synaptic efficacy at both the single-cell and network levels. Furthermore, TDCS generates static electric fields in the brain, whose cellular mechanisms are focused on the neuronal elements activated, such as glial and endothelial cells, as well as Ca^2+^ influx-induced astrocytes [129].

Therefore, it is suggested that this type of synaptic modulation can be used to treat related brain diseases, such as neuropsychiatric diseases and chronic neuropathic pain, in humans [106].

Human participants subjected to TDCS treatment described a tingling sensation in the anatomical regions of the two electrodes during current emission, particularly in the initial stimulation phase. The same tingling sensations have been observed at the cranial level and are considered minimal, and patients should be able to adapt to the sensations within seconds [116, 120].

It seems that motor cortex excitability can modulate pain perception through indirect effects on the thalamic and subthalamic neuroanatomical regions. These factors may be responsible for the inhibition of nociceptive impulses in the spinal cord [120].

In human patients, there is recent evidence suggesting that TDCS combined with TESCS may enhance the effects of robotic-assisted gait training [130]. The use of TDCS with FES should also be considered [131], and the combination of TDCS with conventional locomotor training appears to promote balance recovery. However, there is a need for additional studies on this topic [125].

In our daily veterinary practice, we use a protocol with similar parameters: a current intensity of 1–2 mA; a monophasic/biphasic pulsed rectangular current with ramp-up and ramp-down periods of 30 s; electrode sizes close to 25–35 cm^2^; a session duration of 20 min; and a treatment duration of 5 consecutive days (Figs. 5, 6).

## Conclusions

Both human and small animal patients with SCI can benefit from neurorehabilitation treatment. Dogs and cats that are DPP+ , as well as human patients with ASIA B/C/D impairment, may regain the ability to ambulate with only mild residual proprioceptive deficits with neurorehabilitation treatment.

On the other hand, neurorehabilitation with multidisciplinary protocols is important for dogs and cats that are DPP−, and different approaches, including some electrical stimulation modalities, may help improve flexor–extensor patterns by stimulating a peripheral component (FES) or central component (TDCS and TESCS).

This review was essential for understanding the potential of these modalities in veterinary medicine, addressing three major questions in regard to safety, clinical applications, and the parameters to be used.

Multidisciplinary protocols can benefit from these three electrical stimulation modalities, and the appropriate method can be selected according to the neurologic deficits on an individual basis. Additionally, to increase repetitive training, these protocols are commonly combined with locomotor exercises with an underwater treadmill, land treadmill and/or overground training. However, to determine the effects of these neurorehabilitation modalities in small animals, additional studies are needed.

## Prior publication

Data have not been published previously.

## Authors’ information

AM is a phD student of the Faculty of Veterinary Medicine (University of Lisbon) that began to work in the field of animal physiotherapy in 2007, received a degree from the European School for Advanced Veterinary Studies (ESAVS) and became a Certified Canine Rehabilitation Practitioner (CCRP). Currently, AM is a trainer and instructor for the same institution, affiliated with Tennessee University, as well as an examinator for international exams. AM has contributed to the field of animal rehabilitation in Portugal and given presentations internationally (including Spain, USA, Poland, Germany, Brazil, Sweden and India). AM collaborates with academics in the neurology department at the University of Munich and presents work at many congresses on neurorehabilitation. Additionally, AM is an author of book chapters about the same topics. AM began studying human medicine functional neurorehabilitation in 2015 during two post-graduate opportunities. AM is the clinical director of two major rehabilitation centers in Lisbon and Setubal—CRAL, an ambulatory center with 15–20 patients per week, and CRAA, a hospitalization center with 30–35 patients per week.

DG was a Master’s student of AM in the Faculty of Veterinary Medicine (Lusófona University) and graduated in 2016. DG has studied animal rehabilitation, collaborated on different projects on this field and worked in both rehabilitation centers listed above (CRAA and CRAL) since 2014.

AC graduated from Évora University in 2012. AC has worked with companion animals, especially regarding animal rehabilitation since 2011, and has worked directly with AM at both centers (CRAA and CRAL). Also, has become a CCRP and collaborated with international laboratories in this field and CCRP webinars with special focus on neurorehabilitation.

OG graduated from Faculty of Veterinary Medicine (University of Lisbon) in 2004 and is one of the main responsible for the imaging department of the Veterinary Medicine Scholar Hospital, with focus on neurological imaging.

DM is a recognized university Professor from the University of Tennessee, founder of the CCRP course and an internationally known researcher in the areas of physical therapy and rehabilitation. Also, DM has published in numerous peer-reviewed veterinary journals and is a co-editor of books in the mentioned field.

AF is a full Professor of the Faculty of Veterinary Medicine (University of Lisbon) and Clinical Director of the Veterinary Medicine Scholar Hospital. Also, is an author and co-author of many articles published in international journals, with focus on the fields of neurology and imaging.

## Data Availability

The datasets used and/or analyzed during the current study are available from the corresponding author upon reasonable request.

## Abbreviations

ASIA: American Spinal Injury Association
CPG: Central pattern generator
DPP: Deep pain perception
DPP+: Deep pain perception positive
DPP−: Deep pain perception negative
FES: Functional electrostimulation
FNR: Functional neurorehabilitation
LMN: Lower motor neuron
mA: Milliampere
MS-TESCS: Multisegmental TESCS
RCV/M: Ventral horn/muscle reflex
RST: Reticulospinal descending motor tract
SBBSL: Spinal cord-brainstem-brain-spinal cord loop
SCI: Spinal cord injury
TDCS: Transcranial direct current stimulation
TESCS: Transcutaneous electrical spinal cord stimulation
