# Correction to: Nervous system modulation through electrical stimulation in companion animals

**DOI:** 10.1186/s13028-021-00594-y

**Published:** 2021-08-24

**Authors:** Ângela Martins, Débora Gouveia, Ana Cardoso, Óscar Gamboa, Darryl Millis, António Ferreira

**Affiliations:** 1 Animal Rehabilitation Center, Arrábida Veterinary Hospital, Azeitão, Setúbal Portugal; 2 Faculty of Veterinary Medicine, Lusófona University, Campo Grande, Lisboa, Portugal; 3 Faculty of Veterinary Medicine, University of Lisbon, Lisboa, Portugal; 4 Department of Small Animal Clinical Sciences, University of Tennessee College of Veterinary Medicine, Knoxville, TN USA

## Correction to: Acta Vet Scand (2021) 63:22 10.1186/s13028-021-00585-z

Following the publication of the original article [1], we were notified that on page 6 the authors attribute to Dr. Ammendolia et al. the demonstration of “an increase in blood flow to the spinal cord and cauda equina with TESCS, and the magnitude of effect was dependent on the intensity of the electrical stimulus.” In fact, Dr. Ammendolia and colleagues were citing the original research reported in Budgell BS, Sovak G, Soave D. TENS augments blood flow in somatotopically linked spinal cord segments and mitigates compressive ischemia. Spinal Cord. 2014 Oct;52(10):744–8.

Therefore, reference 131 was added to cite the original work of Budgell et al.

The original article has been corrected.
